# Monoamino Oxidase A Gene Single-Nucleotide Polymorphisms and Methylation Status and the Risk of Violent Suicide Attempts in Affective Disorder Patients

**DOI:** 10.3389/fpsyt.2021.667191

**Published:** 2021-08-02

**Authors:** Birgit Ludwig, Laura Carlberg, Klemens Kienesberger, Patrick Swoboda, Marleen M. M. Mitschek, Alexandra Bernegger, Romina Koller, Michelle Inaner, Birgit Senft, Lisa Meisner, Daniela Fischer-Hansal, Andreas Affenzeller, Jasmin Huber, Silvia Schoenthaler, Nestor D. Kapusta, Helmuth Haslacher, Martin Aigner, Andreas Weinhaeusel, Siegfried Kasper, Alexandra Schosser

**Affiliations:** ^1^Department of Psychiatry and Psychotherapy, Medical University of Vienna, Vienna, Austria; ^2^Department of Neurology, Medical University of Vienna, Vienna, Austria; ^3^Department of Laboratory Medicine, Medical University of Vienna, Vienna, Austria; ^4^Zentren für Seelische Gesundheit, BBRZ-Med, Vienna, Austria; ^5^Molecular Diagnostics Unit, Health and Environment Department, Austrian Institute of Technology, Vienna, Austria; ^6^Department of Psychoanalysis and Psychotherapy, Medical University of Vienna, Vienna, Austria; ^7^Department of Psychiatry and Psychotherapy, Karl Landsteiner University for Health and Science, Tulln, Austria

**Keywords:** suicide, violent suicide, methylation, *Monoamino Oxidase A*, affective disorder

## Abstract

**Background:** When investigating the neurobiology of suicidal behavior, Monoamino Oxidase A (*MAOA*) is one of the prime suspects to consider. Interestingly, *MAOA* dysregulation has also been associated with violent behavior in previous publications. In the present study, we aimed to establish an association between polymorphisms of the *MAOA* gene and methylation status of the *MAOA* gene Exon I, and suicide attempts with violent methods in a sample of affective disorder patients.

**Methods:** Eight hundred fourteen Caucasian affective disorder patients were assessed at the Department of Psychiatry and Psychotherapy of the Medical University Vienna, the Karl Landsteiner University for Health and Science and Zentren für seelische Gesundheit, BBRZ-Med Leopoldau. An assemblage of psychiatric interviews was performed (e.g., SCAN, HAMD, SBQ-R, CTQ) and DNA samples of peripheral blood cells were collected for Sequenom MassARRAY® iPLEX Gold genotyping and Multiplexed and Sensitive DNA Methylation Testing.

**Results:** Female affective disorder patients with a history of violent suicide attempt were found to have a significantly increased frequency of the AA genotype in the rs5906957 single nucleotide polymorphism (*p* = 0.003). Furthermore, the *MAOA* gene exon I promoter region showed significantly decreased methylation in female violent suicide attempter(s) as opposed to female affective disorder patients who had no history of suicide attempt or no history of suicide attempt with violent method.

**Limitations:** The small sample size hampers to reveal small genetic effects as to be expected in psychiatric disorders.

**Conclusions:** This study offers promising findings about associations between the *MAOA* gene and violent suicide especially in women.

## Introduction

Suicide still is a major public health concern. A recent report from the Center for Disease Prevention and Control shows that suicide rates in the US have increased more than 30% in half of the states since 1999 (1). Whereas, total numbers in Austria are slowly diminishing, the suicide rate of 16.4 per 100,000 in 2015 represents a significant public health problem (2, 3). Meanwhile, the suicide mortality rate worldwide reach up to 35.33 per 100,000 population (WHO^1^).

Suicide affects all age ranges, professions, in-patients as well as out-patients, somatic disorders, and mental disorders in particular, and thereof affective disorders show the strongest correlation with suicide (4). Since motives and circumstances of suicide are highly personal and since a myriad of causes can be associated with attempts or completion, suicide really is more the result of complementing biopsychosocial factors than one homogeneous concept. In order to refine this phenotype, attempts to further differentiate suicide into subcategories have been made. Classifying suicide into violent vs. non-violent methods constitutes one of these approaches. Several publications (5, 6) use the Asberg's criteria to identify violent suicide attempts, which defines hanging, the use of firearms, jumping from heights, car crash, burning, gas poisoning, drowning, electrocution, and jumping under a train as violent attempts, whereas drug overdoses are considered to be non-violent suicide attempts (7). Violent suicide has been associated with aggressive personality traits and other predispositions (8), thus a genetic component needs to be taken into consideration.

A myriad of genes and neurotransmitter systems have been associated with violent behavior and suicide, but Monoamino Oxidase A (*MAOA*) gene is one the most extensively investigated in these regards. *Monoamino Oxidase A (MAOA)* is one of the key enzymes in the metabolism of neurotransmitters such as dopamine, serotonin, and norepinephrine. These biogenic monoamines are highly relevant in the pathogenesis of psychiatric disorders and *MAOA* contributes to their degradation by means of oxidative deamination (9). A respectable body of research has been dedicated to associations between suicide attempts and dysregulations of the *MAOA* system (10). Most prominently, the *MAOA VNTR* polymorphism seems to be associated with personality traits of aggression, aggressive behavior, impulsivity, and anti-social behavior (11–15);—in regards to suicidal behavior results are not as conclusive (16–22). Interestingly, on a molecular level, it has been shown that the VNTR genotype did neither influence the methylation status of the gene nor the brain *MAOA* activity (23). Other polymorphisms in the promoter region of the *MAOA* gene have also been investigated. Ouellet-Morin et al. (24) examined the interaction between various *MAOA* polymorphisms (rs5906893, rs5906957, rs2283725, rs2072744, rs979605) and violence in a longitudinal study in male adolescents. The results imply moderating effects of the gene on violent behavior, once a threshold of exposition to violence was reached (24). Similarly, a recent publication investigating single nucleotide polymorphisms (SNPs) of the *MAOA* gene (rs2064070, rs6323, rs909525) revealed sex-specific interactions between personality traits and *MAOA* SNPs in the sample of suicide attempters as compared to healthy controls (25). Furthermore, the Eco RV polymorphism of the *MAOA* gene showed associations with completed suicides in postmortem brain samples of male affective disorder patients (26).

Interestingly, little has been published about the *MAOA* gene and violent suicide. Hung et al. (20) report no association between the uVNTR polymorphism and violent suicide in a sample of psychiatric patients (20). Similarly, no association was found between violent suicides and the uVNTR polymorphism in a Finnish sample of male affective disorder patients (27). Courtet et al.'s (28) results on the other hand suggest that an excess of high-activity *MAOA* gene promoter alleles (uVNTR polymorphism) may be associated with violent suicide (28).

In regards to epigenetic studies, it has been established that peripheral DNA methylation patterns correspond with the brain endophenotype (23). Furthermore, decreased methylation of the first exon seems to be associated with increased gene expression (29). Methylation of the *MAOA* exon I/intron I promoter region has been investigated in a myriad of psychiatric disorders, among them anxiety disorders, depression, posttraumatic stress disorder, antisocial personality disorder, and borderline personality disorder (30). Nonetheless, the present study is to the best of our knowledge the first one to investigate methylation levels of the *MAOA* promoter region in suicide attempters. Two studies from the same lab found decreased methylation of the *MAOA* exon I promoter region in the saliva of female affective disorder patients as compared to female controls. In the first study they tested 92 controls and 82 depressed patients and replicated their results with a smaller sample of 43 controls and 23 depressed patients (31, 32). A recent study from Canada revealed overall hypermethylation of 71 CpG sites in the promoter region of the *MAOA* gene in incarcerated offenders with a diagnosis of antisocial personality disorder when compared to healthy controls. Additionally, they established an interaction model with the significant factors group and single CpG sites (33).

Genetic polymorphisms are known to influence epigenetic mechanisms such as methylation status—but so are life events and lifestyle factors. Thus, investigating both axes is particularly interesting regarding the pathogenesis of psychiatric disorders and the recurring question of state and trait. In this combined candidate gene and methylation study, we aim to identify genetic and epigenetic correlates of violent suicide within a sample of affective disorder patients in an attempt to refine the biopsychosocial model of the pathogenesis of violent suicide.

## Materials and Methods

### Participants

A total of 846 unrelated in- and outpatients with affective disorders were recruited at three study sites in Vienna and the surrounding area. A total of 382 patients were recruited at the Department of Psychiatry and Psychotherapy of the Medical University Vienna, 67 patients Karl Landsteiner University for Health and Science and 397 patients at the Zentren für seelische Gesundheit, BBRZ-Med Leopoldau in the context of the Austrian Science Funds (FWF) funded study “VieSAD” (“Vienna Study on Genetics of Suicidal Behavior in Affective Disorders,” KLI°220). The investigation was carried out in accordance with the latest version of the Declaration of Helsinki and approval for the study was obtained from the Ethical Committee of the Medical University of Vienna (EK 2013/2013) and the federal state of Lower Austria (GS4-EK-4/181/2012).

Caucasians aged from 18 to 65 years were included if they were diagnosed with either bipolar disorder (BD) or major depressive disorder (MDD) as defined by ICD-10 and/or DSM-IV criteria. Exclusion criteria were mood incongruent psychotic symptoms or lifetime history of schizophrenia, primary organic disease, primary substance abuse, pregnancy, and breastfeeding. Diagnosis was affirmed by performing detailed clinical examination [SCAN—Schedules for Clinical Assessment in Neuropsychiatry (34)] and suicidal behavior was assessed by “Viennese Suicide Risk Assessment Scale” (VISURIAS) (35), SBQ-R (Suicidal Behaviors Questionnaire-Revised) (36), and LPC–(Lifetime Parasuicide Count) (37). In order to screen for acute affective states, the HAMD (Hamilton Depression Scale) (38), and MADRS (Montgomery-Asberg Depression Scale) (39) were applied when blood for genotyping was drawn. Biomaterial was processed and stored at the MedUni Wien Biobank facility in an ISO 9001-certified environment according to standard operating procedures published previously (40).

The dichotomous variable previous suicide attempt was created through a total of 12 items from six interview scales (SBQ, MADRS, LPC, HDRS, SCAN, and VISURIAS). To assess a history of previous violent suicide attempt(s), Asberg's criteria (7) were applied. Comorbidities were monitored, as well as weight, height, and Body Mass Index. In a face-to-face interview, patients were informed about the study and signed a written consent form. Interrater reliability was guaranteed by extensive interview training, following Good Clinical Practice criteria.

### Statistical Analyses

All statistics were conducted using the statistical software SPSS 22.0 (IBM, Armonk USA) and “R 3.4.2” (cran.r-project.org/). Continuous data were presented as mean and standard deviation, respectively, with confidence intervals. Categorical data were given as counts and percentages. Fisher's exact test and χ^2^ analysis were calculated to test the equal distribution of categorical variables. Differences between two groups were assessed by means of the Independent Samples *t*-Test. For non-parametric data, Kendall-Tau-b correlation coefficient was calculated. Normal distribution of the variables was tested by Kolmogorov-Smirnov test. All test results were interpreted two tailed with a significance level established at *p* ≤ 0.05. Bonferroni adjustment for multiple comparisons was used for SNPs analyses, and a two-tailed *P*-value of < 0.0125 was taken as statistically significant.

For comparisons of genotype frequencies within the groups of suicidal phenotypes and sex as categorical variables, χ^2^ tests as well as Fisher's exact test (for count < 5) were administered. To test for Hardy-Weinberg equilibrium (HWE), we used online software provided by the Helmholtz Center Munich, applying Pearson's goodness-of-fit chi-square or Fisher's exact test, respectively, (https://ihg.gsf.de/cgi-bin/hw/hwa1.pl). Linkage disequilibrium (LD) analysis was performed using Haploview (41): four SNPs of the MAOA gene were analyzed and frequencies lower than 1% were excluded from the analysis (minor allele frequencies). Power analyses were performed using the software G^*^Power 3.1.9.2; when yielding for a power of ß = 0.80 and assuming an effect size of ω = 0.2, degree of freedom of 2 and α-error probability of 0.05 (42) a sample size of 241 per SNP analysis is needed.

### Sequenom MassARRAY® iPLEX Gold Genotyping

Genomic DNA was isolated from whole blood samples using E.Z.N.A. Blood DNA Mini Kit (Omega bio-tek) according to the manufacturer's protocol. Four SNPs (rs5906957, rs3027399, rs2205718, rs2072744) of the MAOA gene were chosen in accordance with previous literature findings (15, 24, 43–45). Monoamino Oxidase A is located on the short (p) arm of the X chromosome at position 11.3 and the SNPs in the Genome Reference Consortium Human Build 38 are positioned as follows: rs5906957 (A/G): 43688062 on GRCh38 (intron), rs3027399 (C/G): 43733475 on GRCh38 (intron), rs2205718 (G/T): 43738218 on GRCh38 (intron), rs2072744 (C/T): 43740189 on GRCh38 (intron).

The Sequenom MassARRAY® iPLEX Gold assay uses PCR amplification and primer extension, resulting in an allele-specific difference in mass between extension products. The mass difference allows the data analysis software to differentiate between SNP alleles. The iPLEX Gold assay has been used in hundreds of publications to routinely design assays at a multiplexing level up to 40-plex, and up to 384 samples can be processed in parallel.

Sequenom Genotyping was performed at the Division of Genetic Epidemiology, Department of Medical Genetics, Molecular and Clinical Pharmacology of the Medical University of Innsbruck (Schöpfstrasse 41, 6020 Innsbruck, Austria) in cooperation with Dr. Stefan Coassin. A total of five samples had to be excluded due to DNA concentration < 1 ng/μl. The average call rate of the analysis was >97%. Cluster plots were checked manually and quality control of the raw data did not show any abnormalities.

### Methylation Analysis

Epigenetic methylation analyses were performed using the MSRE (methylation-sensitive restriction enzyme)—qPCR (quantitative polymerase chain reaction) approach (46). For methylation analyses, based on previous literature, the CpG island covering part of the *MAOA* gene Exon I was selected (chrX:43,654,925-43,655,084, hg38) with a size of 160 bp (PCR primers—forward: tagagtcacttctccccgcccctga and reverse: gctgaggcgtttgtgctcatgttt), containing a total of 10 CpG sites; of those 6 CpGs were covered by the methylation sensitive restriction enzymes AciI, HpaII, Hin6i, and HpyCH4VI.

Digestion of all samples and parallel incubation without digestion was performed the same day. Two microliters of digested/undigested DNA was used for the following qPCR protocol: hotstart activation with 95°C for 5 min, followed by amplification (45x) with 95°C for 40 s, 65°C for 40 s, 72°C for 1 min 20 s, followed by the final extension step with 72°C for 7 min and cool-down phase with 4°C. Raw data of methylation analyses (Ct- and Tm-values) were calculated. Methylation status in % was calculated relatively to the reference values of undigested DNA samples applying the following formula:

% methylation = sample digested [ng] / sample undigested [ng] ^*^ 100%

The according values were reported as Percent methylated reference (PMR) values.

## Results

In total, 846 patients were recruited, 32 patients had to be excluded from further analyses because of incomplete interview data or presence of exclusion criteria, resulting in a total of 814 patients. The mean age of the participants was 44.6 years (*SD* = ±12.9). A total of 34.8% of the patients had a history of suicide attempt(s) and 10.7% had a history of violent suicide attempt(s). Further descriptive statistics combining diagnoses, sex, and history of suicide attempt of the sample are presented in Supplementary Table 1.

### Genotyping

The primary goal was to test for associations of *MAOA* SNPs with (history of) violent suicide attempt(s) in a cohort of affective disorder patients. Since the *MAOA* gene is located on the X-chromosome, analyses were carried out separately for female and male subjects.

The female subsample (*n* = 535) was investigated in regards to associations between previous violent suicide attempt(s) and HWE, genotypes, alleles, and haplotypes. No significant HWE deviations were found for the female controls [no history of violent attempt(s)]; two SNPs of the previous violent suicide attempt group showed significant HWE deviation: rs2072744 (*p* = 0.037) and rs5906957 (*p* = 0.01). Lifetime history of violent suicide attempt (yes/no) was analyzed as a dichotomous trait applying the standard chi-square statistics (and Fisher's exact test for the SNPs with allele frequency < 5) finding no allelic associations but revealing a significant genotype association in SNP rs5906957 (χ^2^ = 11.81, *p* = 0.003—withstanding Bonferroni correction for multiple testing; see Table 1). The selection criteria for haplotypes were adjacent SNPs with pairwise *r*^2^ > 0.80. LOD threshold for multi-marker tests was ≥3. Only haplotypes with frequencies above 0.01 were tested. According to the selection criteria, three SNPs (rs302739, rs2205718, and rs2072744) with strong *r*^2^ > 0.80 were in one block (Supplementary Figure 1). Further association analysis for the adjacent block did not reveal any significant associations between any of the haplotypes (G-G-C/G-T-T/C-G-C/G-G-T) and lifetime history of violent suicide attempt(s) in the female subsample (Supplementary Table 2).

**Table 1:** Female subsample: personal history of violent suicide attempt vs. no history of violent suicide attempt, single marker analyses were established with standard chi-squared testing.

**MAOA**			**Genotypes**		**Alleles**		**HWE**	
**SNP ID**	**Violent suicide attempt**	**No violent suicide attempt**	**χ^**2**^**	***p***	**χ^**2**^**	***p***	**Violent suicide attempt**	**No violent suicide attempt**
rs5906957	42 (AA: 9, GA: 12, GG: 21)	397 (AA: 26, GA: 156, GG: 215)	11.81	0.003	3.38	0.07	*p* = 0.01	*p* = 0.75
rs3027399	43 (CC: 1, CG: 5, GG: 37)	405 (CC: 2, CG: 38, GG: 365)	2.83^*^	0.24	1.22	0.27	*p* = 0.29^*^	*p* = 0.23^*^
rs2205718	41 (GG: 18, GT: 15, TT: 8)	397 (GG: 197, GT: 173, TT: 45)	2.49	0.29	1.21	0.27	*p* = 0.16	*p* = 0.44
rs2072744	38 (CC: 17, TC: 12, TT: 9)	373 (CC: 163, TC: 164, TT: 46)	4.57	0.10	0.77	0.38	*p* = 0.037	*p* = 0.63

*MAOA gene, monoamino oxidase A gene; SNP, single nucleotide polymorphism; χ^2^, Pearson's chi-squared test, HWE, Hardy-Weinberg-equilibrium*.

* *Calculated with Fisher's Exact Test*.

In the male subsample (*N* = 279), no significant associations for genotype/allele frequencies were revealed when comparing cases [history of violent suicide attempt(s)] and controls [no history of violent suicide attempt(s); Supplementary Table 3].

In order to establish if the *MAOA* gene was generally associated with suicide in females, we also tested for previous suicide attempt(s) (irrespective of the method). In the female subsample, no associations were found between MAOA genotypes, alleles, haplotypes, and previous suicide attempt(s). There were no significant HWE deviations, neither in cases [previous suicide attempt(s)] nor in controls [no previous suicide attempt(s)] in the female subsample (Table 2).

**Table 2:** Personal history of suicide attempt(s) vs. no history of suicide attempt in female affective disorder patients, single marker analyses were established with standard chi-squared testing.

			**Genotypes**		**Alleles**		**HWE**	
**SNP ID**	**Previous suicide attempt**	**No previous suicide attempt**	**χ^**2**^**	***p***	**χ^**2**^**	***p***	**Previous suicide attempt**	**No previous suicide attempt**
rs5906957	129 (AA: 16, GA: 47, GG: 66)	311 (AA: 20, GA: 121, GG: 170)	4.33	0.12	1.99	0.16	*p* = 0.11	*p* = 0.81
rs3027399	131 (CC: 1, CG: 9, GG: 121)	318 (CC: 2, CG: 34, GG: 282)	1.75^*^	0.43^*^	1.06	0.30	*p* = 0.20	*p* = 0.31
rs2205718	127 (GG: 53, GT: 57, TT: 17)	312 (GG: 144, GT: 131, TT: 37)	0.74	0.69	0.69	0.41	*p* = 0.79	*p* = 0.39
rs2072744	120 (CC: 49, TC: 54, TT: 17)	292 (CC: 131, TC: 122, TT: 39)	0.56	0.75	0.41	0.52	*p* = 0.73	*p* = 0.22

*MAOA gene, monoamino oxidase A gene; SNP, single nucleotide polymorphism; χ^2^, Pearson's chi-squared test; HWE, Hardy-Weinberg-equilibrium*.

* *Calculated with Fisher's Exact Test*.

### Methylation Analysis

**Figure 1:**
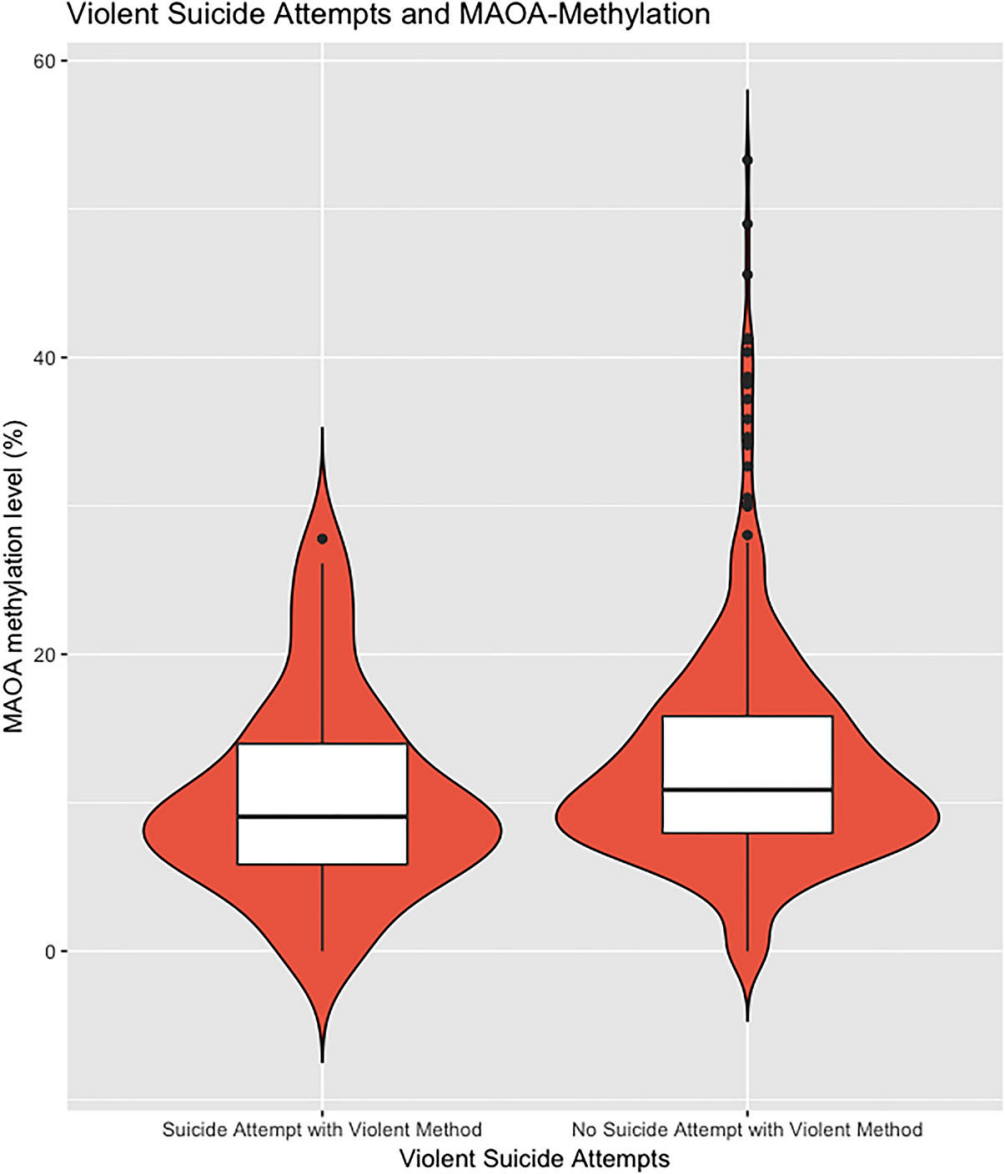
The extent of methylated DNA at MAOA exon I promoter in peripheral blood of female affective disorder patients with a history of violent suicide attempt(s) (10.31 ± 6.61%; *n* = 51) compared to female patients without any history (13.12 ± 11.93%; *n* = 476; *p* = 0.01; t-test). Violin plot with median and interquartile range.

**Figure 2:**
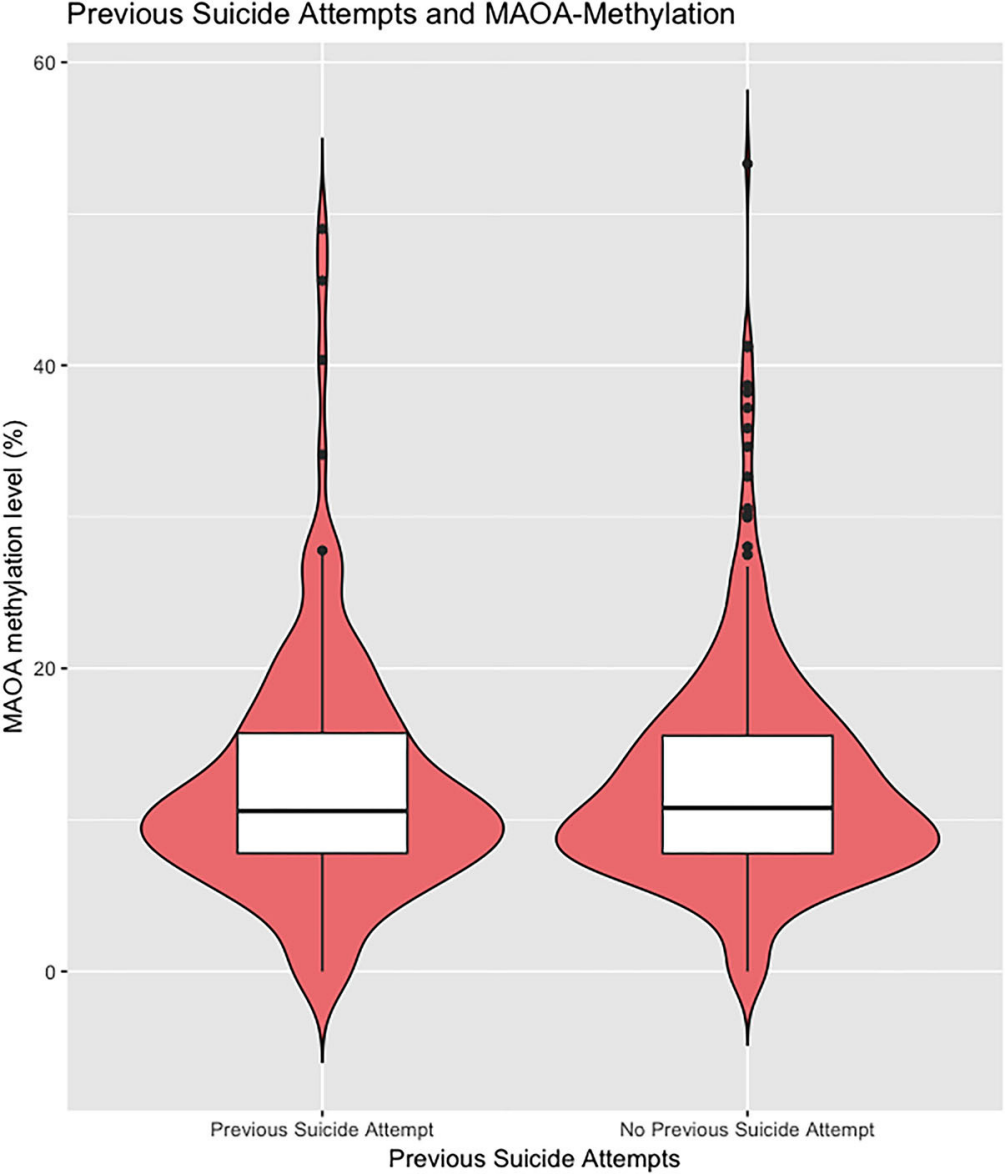
The extent of methylated DNA at MAOA exon I promoter in peripheral blood of female affective disorder patients with and without a personal history of suicide attempt(s) (previous attempt: 14.48 ± 13.83%, *n* = 138; no previous attempt: 13.50 ± 10.31%, *n* = 356; *p* = 0.39, *t*-test). Violin plot with median and interquartile range.

To examine the methylation status of *MAOA* exon I promoter region, the MSRE–qPCR approach was performed. A total of 757 samples were successfully analyzed. As to be expected, there was a significant difference in the methylation status of the *MAOA* exon 1 promoter region between male and female affective disorder patients (men: 0.67 ± 2.32%; *n* = 268; women: 13.51± 11.40%, *n* = 507; *p* < 0.001, *t*-test; see Supplementary Figure 2). Hence, all analyses were performed separately for both subsamples. Female affective disorder patients with a history of violent suicide attempt(s) (10.31 ± 6.61%; *n* = 51) showed decreased methylation levels compared to female patients without any history (13.91 ± 11.83%; *n* = 449; *p* = 0.03; *t*-test) (Figure 1). As for male affective disorder patients, there was no significant difference in methylation levels between patients with (0.49 ± 1.50%; *n* = 35) and without history of violent suicide attempts (0.70 ± 2.44%; *n* = 229; *p* = 0.61, *t*-test) (Supplementary Figure 3). When testing for previous suicide attempts, irrespective of the method, there was no difference found neither in the male (previous attempt: 0.74 ± 2.32%, *n* = 66; no previous attempt: 0.65 ± 2.34%, *n* = 200; *p* = 0.79, t-test) (Supplementary Figure 4) nor in the female sample (previous attempt: 14.17 ± 13.84%, *n* = 141; no previous attempt: 13.31 ± 10.34%, *n* = 361; *p* = 0.45, *t*-test) (Figure 2).

**Figure 3:**
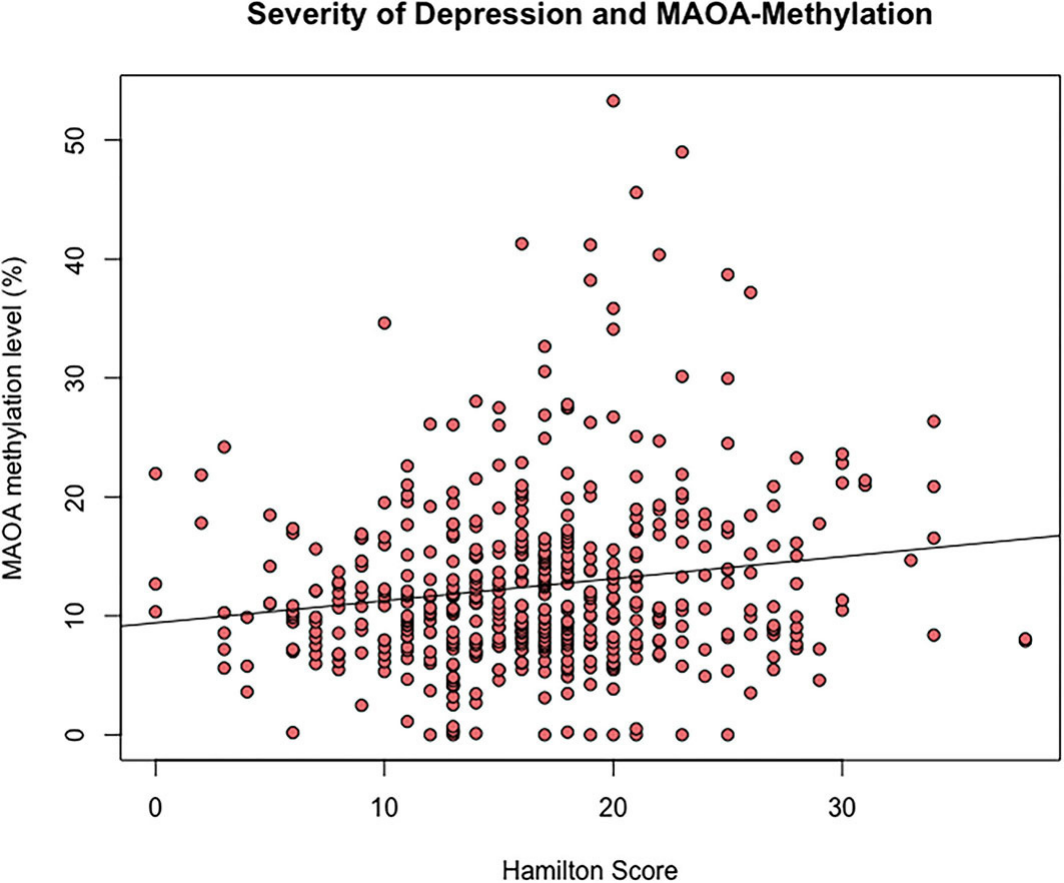
Scatterplot of Hamilton Depression Score and MAOA gene promoter region exon I methylation (%) of female affective disorder patients (τ = 0.12, *p* < 0.001, *n* = 535).

In the female subsample, a positively significant but weak correlation was found for PMR values and the total Hamilton Depression (HAMD) score (*n* = 497), as inferred from the Kendall-Tau-b correlation coefficient (τ = 0.11, *p* < 0.001) (Figure 3). In the male subsample, there was no significant correlation between methylation status of *MAOA* and the total HAMD score in this sample of male affective disorder patients (*n* = 264, τ = 0.01, *p* = 0.82) (Supplementary Figure 5).

The aim of this study was to find correlations with clinical parameters but in order to account for interactions between genetic and epigenetic variations, one-way ANOVA was performed for methylation status and SNPs. Significant results are illustrated in Supplementary Figures 6–9.

## Discussion

In this study we were aiming to establish an association between polymorphisms and the methylation status of the MAOA gene, and suicide attempts with violent methods in a sample of affective disorder patients. As it has thoroughly expounded in the introduction part, both violent behavior and suicide have been associated with dysregulation of the MAOA gene, hence it being combined in violent suicide attempt(s) generates a promising hypothesis to test for.

The main findings of the present study show a dysregulation of the MAOA gene, both on a genetic as well as on an epigenetic level, in female affective disorder patients with a personal history of violent suicide attempt(s). Female affective disorder patients with a personal history of violent suicide attempt(s) seem to have an increased frequency (0.21 in cases vs. 0.065 in controls) of the AA genotype in the SNP rs5906957 (χ^2^ = 11.81, *p* = 0.003—withstanding Bonferroni correction for multiple testing). The main finding in the methylation analysis points toward a similar direction; the *MAOA* gene exon I promoter region showed decreased methylation in female violent suicide attempter(s) as opposed to female affective disorder patients who did not attempt suicide or did not attempt suicide with violent methods (*p* = 0.01; *t*-test). It could be hypothesized, that the present hypomethylation of CpGs in Exon I would be associated with increased *MAOA* gene expression (29) in these female affective disorder patients.

The significant sex differences in methylation levels of the *MAOA* gene was to be expected, since it is located on the X chromosome (hence men only have one allele). Our results replicate previous findings in the saliva of female affective disorder patients showing hypermethylation of the *MAOA* exon I promoter compared to male affective disorder patients (32). This hypermethylation in females is suspected to be the mechanism behind X-chromosome inactivation of one of the two *MAOA* alleles (47).

The present results are suggesting that not previous suicide attempts in general are associated with the *MAOA* system but only attempts with violent methods in female affective disorder patients. Interestingly, this association could be demonstrated both on a genetic as well as on an epigenetic level. The observed decreased methylation in the violent attempt sample might be associated with increased expression of *MAOA* in the brain and concomitant decreased availability of monoamines such as serotonin, dopamine, and norepinephrine in the synaptic cleft, as suggested by *in vitro* findings (33). Hence, increased serotonin levels in the brain of depressed individuals might account for a more aggressive or violent approaches in attempting suicide. The question why the tested hypothesis only withstood in female affective disorder patients remains unclear and further studies will be needed to illuminate the topic.

Limitations of this study include a small sample size to reveal small genetic effects as expected to be involved in the pathogenesis of suicidal behavior. Aside from that the assessment of suicidality was performed in a retrospective fashion and although multiple items from various interview scales were combined to create the variables, the accuracy of reported suicide attempts cannot be guaranteed. Another important limitation regarding the informative value of this study is the selection of SNPs of the *MAOA* gene; we did not include the ubiquitously studied VNTR polymorphism in our selection of SNPs but selected SNPs further downstream based on a tag SNP approach. Regarding the methylation analysis, in female samples a theoretical 50% methylation value would be expected, due to X–inactivation. Using the MSREqPCR approach, we obtained lower methylation values then the expected 50% value. A potential explanation for the lack of the “theoretical 50% value” might be due to the presence of unmethylated single CpGs in the inactive X-chromosome within the studied 160 bp *MAOA* region; these CpGs representing MSRE cut-sites are then digested and not PCR amplified, thus causing the deviations from 50% theoretical values.

One of the clear strengths of this study is the precise phenotypic definition of our sample, which results in a highly homogeneous sample and allows us to prevent interference of the confounding affective disorders phenotype. Another strength of this study is the large sample size of the methylation analysis (757 affective disorder patients), compared to previous studies.

In conclusion, the herein presented results support the hypothesis that the *MAOA* gene is involved in violent suicide attempts in female affective disorder patients. To the best of our knowledge the current study is the first one to investigate these SNPs and methylation levels of the *MAOA* gene exon I promoter region in affective disorder patients with a lifetime history of suicide attempts. Needless to say, that the risk of attempting suicide with violent methods is influenced by a myriad of factors, among them not only neurobiological but equally psychological and social implications. Nonetheless, our findings are mostly in line with previous findings—linking the implication of aggression and violent behavior with suicide attempts. A larger sample size, broader SNP selection and replication of the current findings are necessary to ultimately elucidate the link between violent suicide and dysregulation of the *MAOA* system.

## Data Availability Statement

The datasets presented in this study can be found in https://mfr.osf.io/render?url=https%3A%2F%2Fosf.io%2F2kjva%2Fdownload.

## Ethics Statement

The studies involving human participants were reviewed and approved by Ethical Committee of the Medical University of Vienna (EK 2013/2013) and the federal state of Lower Austria (GS4- EK-4/181/2012). The patients/participants provided their written informed consent to participate in this study.

## Author Contributions

All authors have made a substantial contribution either to the conception and design, or to the acquisition of data, or to the analysis and interpretation of the data, have made a substantial contribution to drafting the article or reviewing it critically, and have given final approval of the version of the article to be published.

## Conflict of Interest

SK received grants/research support, consulting fees, and/or honoraria within the last three years from Angelini, AOP Orphan Pharmaceuticals AG, Celegne GmbH, Eli Lilly, Janssen-Cilag Pharma GmbH, KRKA-Pharma, Lundbeck A/S, Mundipharma, Neuraxpharm, Pfizer, Sanofi, Schwabe, Servier, Shire, Sumitomo Dainippon Pharma Co. Ltd., and Takeda. The remaining authors declare that the research was conducted in the absence of any commercial or financial relationships that could be construed as a potential conflict of interest.

## Publisher's Note

All claims expressed in this article are solely those of the authors and do not necessarily represent those of their affiliated organizations, or those of the publisher, the editors and the reviewers. Any product that may be evaluated in this article, or claim that may be made by its manufacturer, is not guaranteed or endorsed by the publisher.

## Footnotes

^1^World Health Organization (WHO). *Suicide*. Fact sheet. Available online at: http://www.who.int/mediacentre/factsheets/fs398/en/ (updated March 2017).
