# Supplementary material for: Monoamino Oxidase A Gene Single-Nucleotide Polymorphisms and Methylation Status and the Risk of Violent Suicide Attempts in Affective Disorder Patients
Source: Front Psychiatry. 2021 Aug 2;12:667191. doi: 10.3389/fpsyt.2021.667191 (PMC8378401; doi:10.3389/fpsyt.2021.667191)
Supplement: Supplementary file 1 [file Data_Sheet_1.pdf]

# *Supplementary Material*

| | Female (n=535) | | Male (n=279) | | Whole Sample (n=814) | |
|---|---|---|---|---|---|---|
| MDD | n = 478 | 89.3% | n = 238 | 85.3% | n = 716 | 88.0% |
| BD | n = 57 | 10.7% | n = 41 | 14.7% | n = 98 | 12.0% |
| Suicide Attempt | n = 149 | 27.9% | n = 67 | 24% | n = 216 | 26.5% |
| Violent Suicide Attempt | n = 51 | 9.5% | n = 36 | 12.9% | n = 87 | 10.7% |

**Supplementary Table 1:** Descriptive statistics of diagnosis and suicidal behavior separated by sex (n = number)

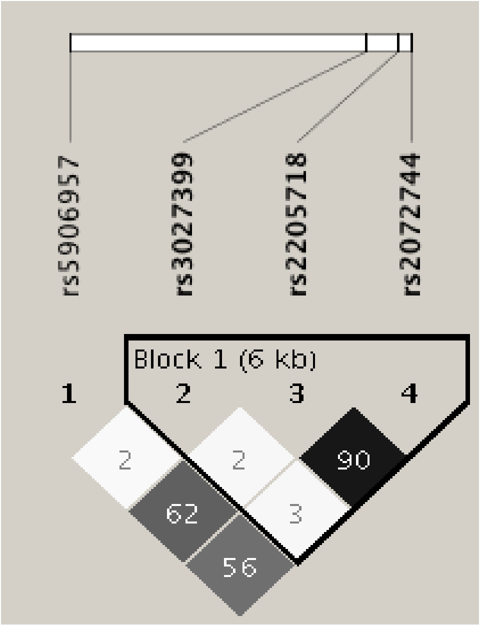


**Supplementary Figure 1:** Linkage disequilibrium (LD) analysis of female affective disorder with violent suicide attempt. LD analysis was performed using Haploview software. The selection criteria for haplotypes used in the analyses were adjacent SNPs with pairwise r2>0.80; also only haplotypes with frequencies above 0.01 were tested. According to the selection criteria, three of the four tested SNPs of the MAOA gene (rs307399- rs2205718-rs2072744) were in one block.

| No. of Markers | Haplotype | Frequency | Alleles increasing in cases | Estimated haplotype frequency % | | $\chi 2$ | p-value |
|---|---|---|---|---|---|---|---|
| | | | | Female patients with previous violent suicide attempts | Female patients without previous violent suicide attempts | | |
| 3 | rs307399-rs2205718-rs2072744 | 0.58 | G-G-C | 0.53 | 0.59 | 1.23 | 0.27 |
| 3 | rs307399-rs2205718-rs2072744 | 0.34 | G-T-T | 0.37 | 0.34 | 0.31 | 0.58 |
| 3 | rs307399-rs2205718-rs2072744 | 0.05 | C-G-C | 0.08 | 0.05 | 1.30 | 0.25 |
| 3 | rs307399-rs2205718-rs2072744 | 0.02 | G-G-T | 0.03 | 0.02 | 0.03 | 0.87 |

**Supplementary Table 2:** Estimated frequency of haplotypes and association significance: Chi-Square values and Pearson's p-values (global and individual) and Odds Ratio with 95% Confidence Interval in a subsample comparing female patients with previous violent suicide attempts with female patients without previous violent suicide attempts ($\chi 2$ = Pearson's Chi-squared test, No = number)

| MAOA | | | Genotype/Allele Frequencies | |
|---|---|---|---|---|
| SNP ID | Violent Suicide Attempt | No Violent Suicide Attempt | χ2 | p |
| rs2072744 | 29 (C: 18, T: 11) | 175 (C: 119. T: 56) | 0.40 | 0.53 |
| rs5906957 | 31 (A: 8, G: 23) | 188 (A: 43, G: 145) | 0.13 | 0.72 |
| rs2205718 | 31 (G: 22, T: 9) | 188 (G: 138, T: 50) | 0.08 | 0.78 |
| rs3027399 | 32 (C: 3, G: 29) | 191 (C: 13, G: 178) | 0.27 | 0.71* |

**Supplementary Table 3:** Male subsample: Personal history of Violent Suicide Attempt vs. no history of Violent Suicide Attempt. single marker analyses were established with standard chi-squared testing (MAOA gene = Monoamino Oxidase A gene. SNP = Single Nucleotide Polymorphism. $\chi^2$ = Pearson's Chi-squared test. HWE = Hardy-Weinberg-Equilibrium). * calculated with Fisher's Exact Test

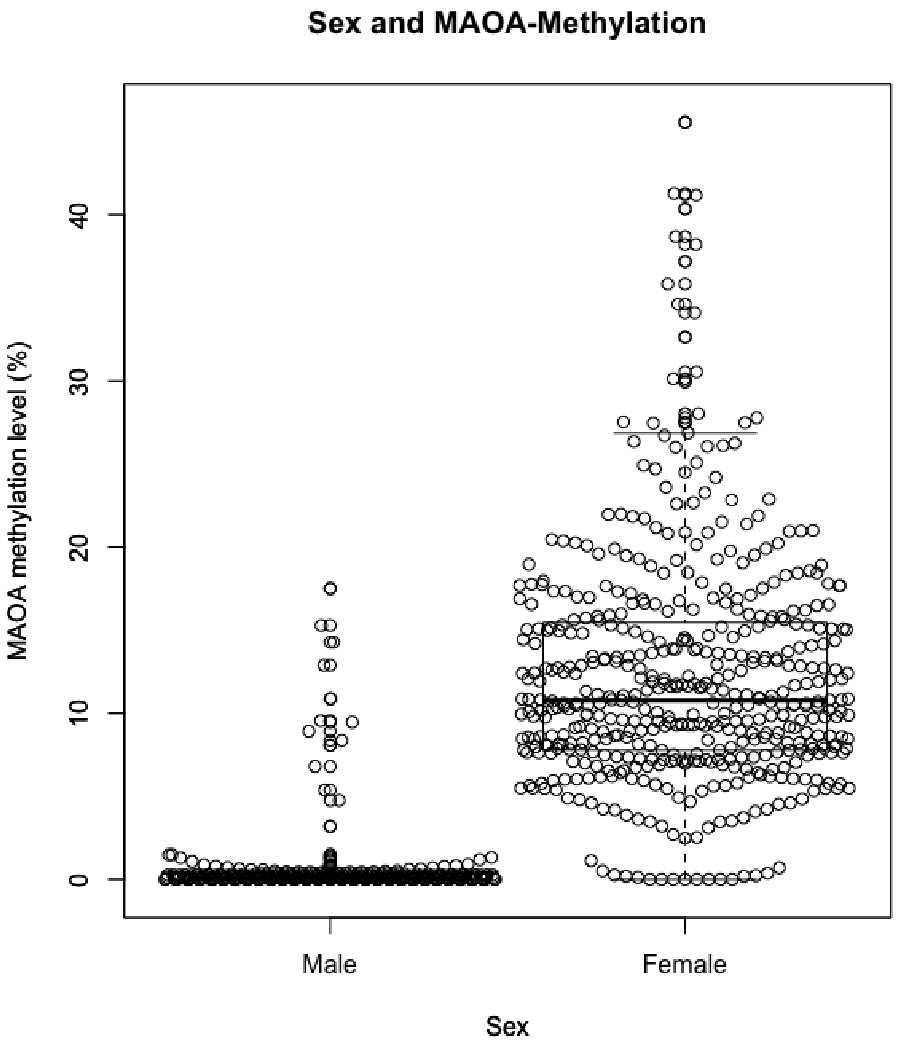


**Supplementary Figure 2:** The extent of methylated DNA at MAOA exon I promoter in peripheral blood of male and female affective disorder patients (men: 0.64 ± 2.26%; n=279; women: 12.80 ± 11.50%, n=535; p < 0.001, t-test). Boxplot with median and whiskers.

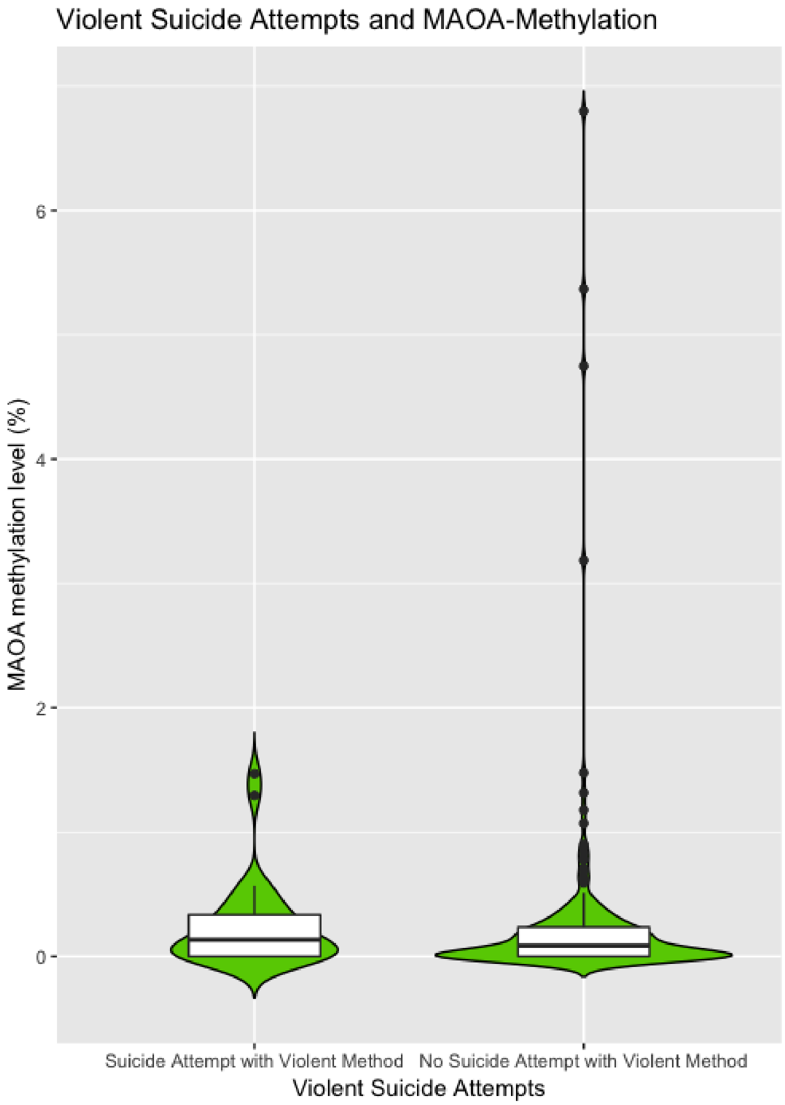


**Supplementary Figure 3:** The extent of methylated DNA at MAOA exon I promoter in peripheral blood of male affective disorder patients with (0.24 ± 0.34; n=34) and without history of violent suicide attempts (0.25 ±0.72; n=220; p=0.93, t-test)

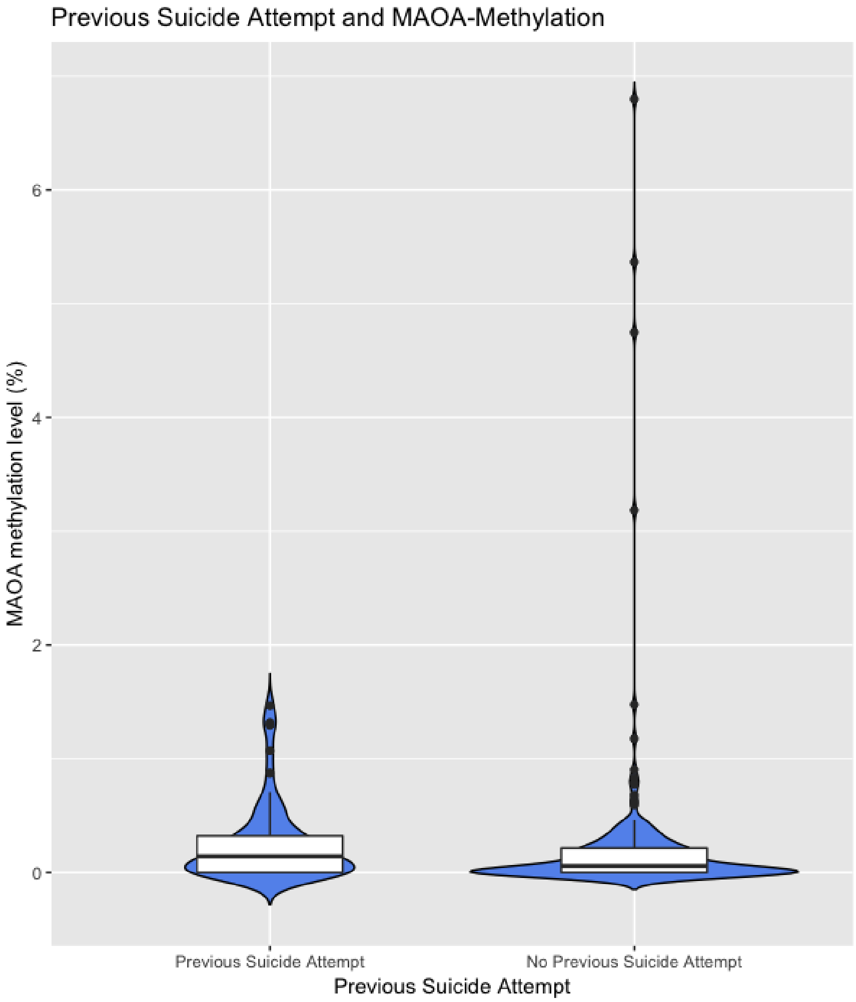


**Supplementary Figure 4:** The extent of methylated DNA at MAOA exon I promoter in peripheral blood of male affective disorder patients with (0.74 ± 2.32%, n=66) and without history of violent suicide attempts (0.65 ± 2.34%, n=200; p=0.79, t-test)

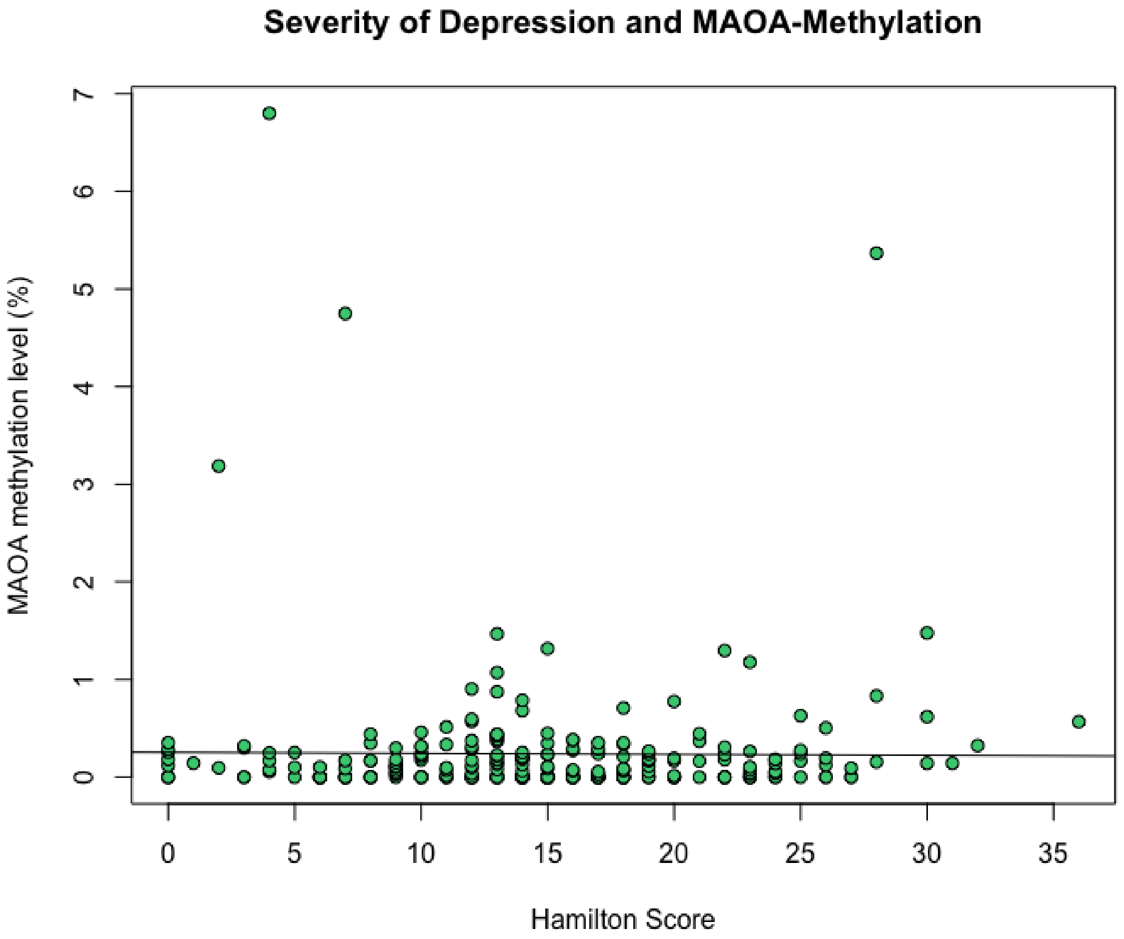


**Supplementary Figure 5:** Scatterplot of Hamilton Depression Score and MAOA gene promoter region exon I methylation (%) of male affective disorder patients (n=275, $\tau = 0.006$, p=0.90).

# Methods Supplementary Material – description of methylation analysis:

Epigenetic methylation analyses were performed at the Austrian Institute of Technology GmbH, Center for Health and Bioresources (Giefinggasse 4, 1210 Vienna), in cooperation with Priv.-Doz. DI Dr. Andreas Weinhäusel (Thematic Coordinator Molecular Diagnostics), using the MSRE (methylation-sensitive restriction enzyme) – qPCR (quantitative polymerase chain reaction) approach. For methylation analyses, based on previous literature, the MAOA (monoamino oxidase A) gene Exon I was selected (chrX:43654783-43655545) with a size of 763 alleles, containing a total of 54 CpG sites.

Digestion of all probes was performed the same day, and parallel incubation without digestion (that is within the reaction buffer but without enzyme) was performed. Incubation time was 16 hours at 37° Celsius, 50 minutes at 65° Celsius. The digestion preparation included 3µl buffer Tango, 0.4µl Aci I (10U/µl), 0.4µl Hin 6l (10U/µl), 0.4µl HpyCHVIV (10U/µl), 17.9µl aqua dest, and 7.5µl DNA (100ng).

For performing qPCR 10µl with 0.5µl DMSO was chosen, and 2µl digested/undigested DNA was added. PCRs were performed on 384-well plates, and 2 DNA standard dilution rows were placed on each PCR plate. The protocol for qPCR was as follows: hotstart activation with 95°C for 5min, followed by amplification (45x) with 95°C for 40sec, 65°C for 40sec, 72°C for 1min 20sec, followed by the final extension step with 72°C for 7min and cool-down phase with 4°C.

Raw data of methylation analyses (Ct- and Tm-values) were imported into Microsoft Excel for further analyses, and medians of Tm-values of DNA standards were calculated. The following formula was used for Tm-reassessment of the Ct-values:

*=IF(AND(„Tm-value of the samples"<="Tm-value median"+1,5;"Tm-value of the samples">="Tm-value median"-1,5);"ct-value of the samples";"")*

The Ct-value of a sample will be adopted if the accompanying Tm-value is within a range of +/- 1.5°C of the previously calculated median of the Tm-values of the standard. If the Tm-value is without this range, a cell remains empty.

Using the Ct-values of the standard row, the parameters gradient, axis intercept, efficacy and coefficient of determination were calculated. In a diagram, DNA-concentrations in ng per reaction (y-axis) were plotted against Ct-values (x-axis), with a logarithm scale of the 3 standards illustrated separately. Of the three linear equations resulting from the separating line, the "best" according to coefficient of determination and calculated PCR efficacy was chosen, and used for calculation of the DNA contents of the digested samples. The DNA content of the undigested reference sample was calculated applying this procedure as well. Methylation status in % was calculated relatively to the reference values applying the following formula:

*% methylation = sample digested [ng] / sample undigested [ng] * 100%*

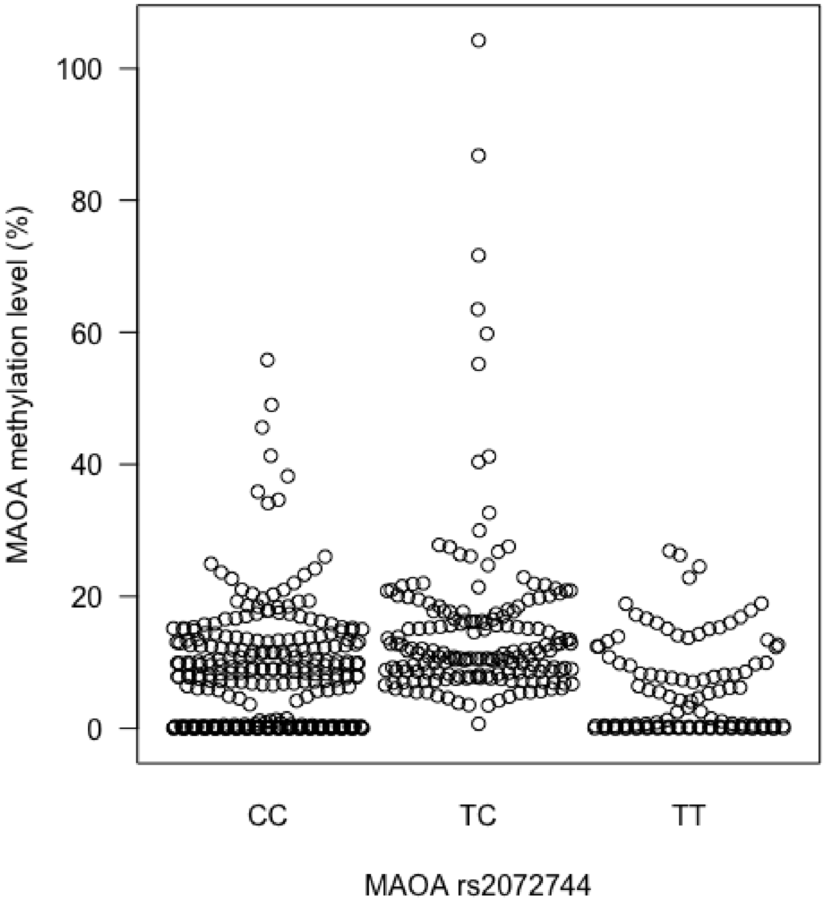


**Supplementary Figure 6:** Overall one-way ANOVA between the 3 genotypes was: $F = 47.8$, $df = 2$, $p < 0.001$; $p < 0.001$ when TC group was compared with CC; $p < 0.001$ when TC was compared to TT; $p = 0.134$ when CC and TT were compared with each other.

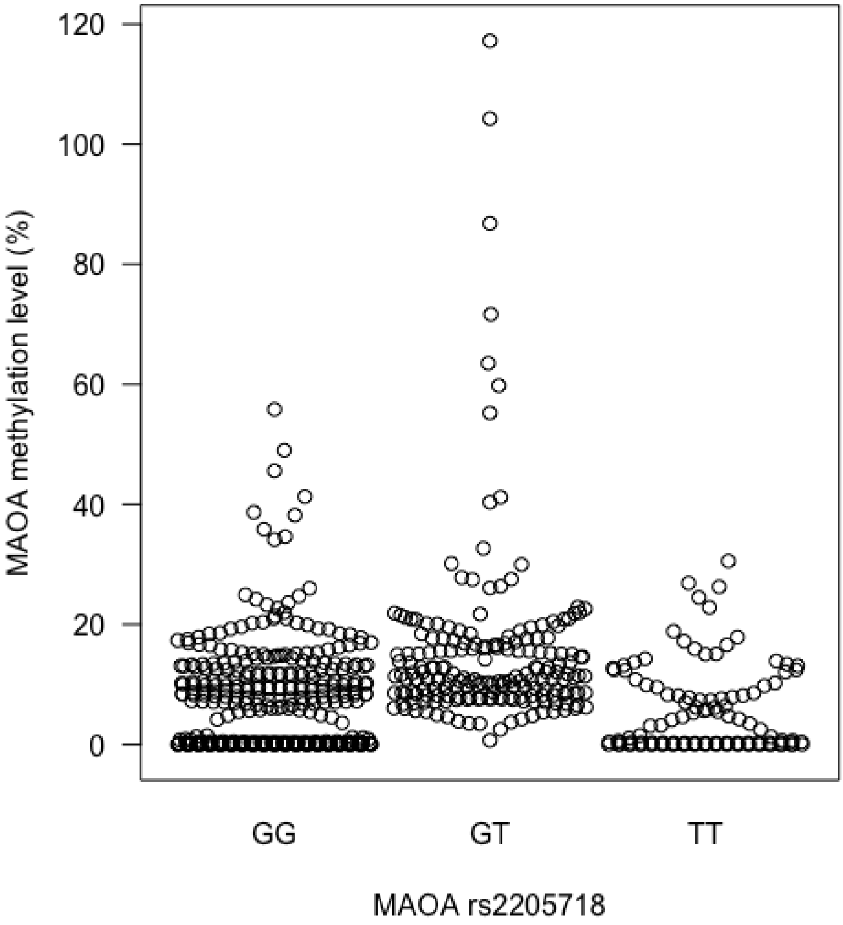


**Supplementary Figure 7:** Overall one-way ANOVA between the 3 genotypes was: $F = 46.3$, $df = 2$, $p < 0.001$; $p < 0.001$ when GT group was compared with GG; $p < 0.001$ when GT was compared to TT; $p = 0.298$ when GG and TT were compared with each other.

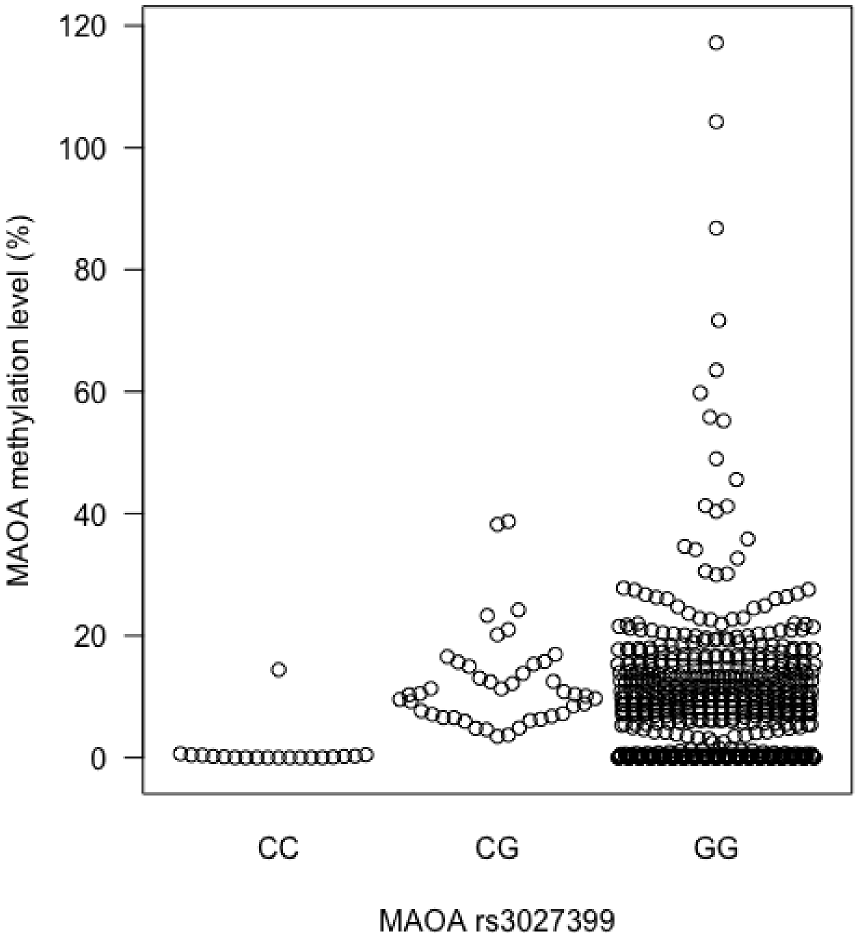


**Supplementary Figure 8:** Overall one-way ANOVA between the 3 genotypes was: F = 6.9, df = 2, p = 0.001; p = 0.001 when CC group was compared with GG; p = 0.004 when GG was compared to CG; p = 0.279 when CG and CC were compared with each other.

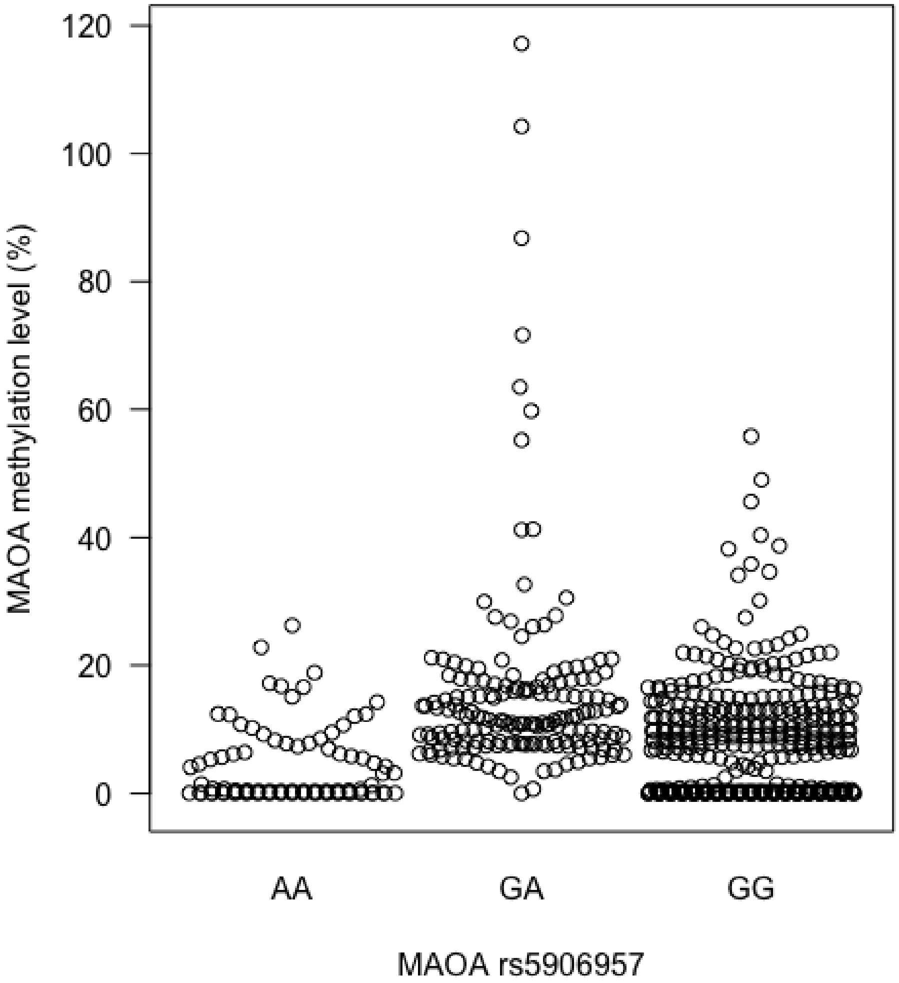


**Supplementary Figure 9:** Overall one-way ANOVA between the 3 genotypes was: F = 44.1, df = 2, p < 0.001; p < 0.001 when AA group was compared with GA; p < 0.001 when GA was compared to GG; p = 0.018 when AA and GG were compared with each other.
